# Characterization of puma–livestock conflicts in rangelands of central Argentina

**DOI:** 10.1098/rsos.170852

**Published:** 2017-12-06

**Authors:** María de las Mercedes Guerisoli, Estela Luengos Vidal, Marcello Franchini, Nicolás Caruso, Emma Beatriz Casanave, Mauro Lucherini

**Affiliations:** 1 GECM (Grupo de Ecología Comportamental de Mamíferos), Lab. de Fisiología Animal, Depto. Biología, Bioquímica y Farmacia, UNS (Universidad Nacional del Sur), Bahía Blanca, Argentina; 2 INBIOSUR (Instituto de Ciencias Biológicas y Biomédicas del Sur), CONICET, UNS (Universidad Nacional del Sur)—DBByF (Departamento de Biología, Bioquímica y Farmacia), San Juan 670, Bahía Blanca, Argentina; 3 Department of Bioscience, University of Parma, Parma, Italy

**Keywords:** Argentinean Espinal, carnivores, interviews, livestock predation, mitigation, *Puma concolor*

## Abstract

Livestock predation is one of the major causes of conflicts between humans and pumas (*Puma concolor*). Using data from interviews with ranchers and kill-site inspections, we characterized puma–livestock conflicts in Villarino and Patagones counties of central Argentinean rangelands. Depredation was considered the major cause of livestock losses, and puma attacks were reported in 46.6% and 35.4% of ranches in Villarino and Patagones, respectively. The majority of ranches underwent losses smaller than 1000 USD. The proportion of livestock lost to predation (0.1–10.4%) and financial losses (5.3–1560.4 USD) per ranch/year varied across ranches, and small sheep ranches in Villarino were affected the most. Depredation was recorded only at night and preferentially in grassland with shrubs and cropland habitats. Although nocturnal enclosures appeared to decrease sheep losses, puma hunting was considered the most effective form of reducing depredation and was implemented by most ranchers. Mortality rates were 3.7 and 1.1–1.56 individuals/year × 100 km^2^ for sheep and pumas, respectively. Nocturnal fencing, shepherding and spatial separation from predators may efficiently reduce sheep losses. However, the poor association between the intensity of puma persecution and puma-related livestock losses suggests that conflict mitigation in central Argentina is not only about reducing damage but also about increasing tolerance.

## 1. Introduction

Most large carnivores have undergone marked declines in both population size and geographical range and because of increasing encroachment with human activities, the maintenance of viable populations across much of their range ultimately depends upon mitigating conflict with humans [1]. Predation on livestock is viewed as one of the major causes of this conflict and consequently, a major driver of the depletion of large carnivore populations worldwide [2–4]. Predicting the outcomes of carnivore–human conflicts for both components of this equation may be challenging, because it requires assessment of human responses, as well as those of the carnivores [5]. However, this knowledge is critical because an increasing proportion of carnivore populations lives entirely within productive agricultural and ranching systems and their survival is almost completely dependent on a sustainable coexistence with humans [4–7].

Pumas (*Puma concolor*) are thought to perform a regulatory function in ecosystems by influencing prey and smaller predator behaviours and population abundances [8,9]. In spite of this important role in ecosystems and of the fact that this felid is the wild carnivore with the largest distribution in the Americas, puma ecology in South America is still relatively understudied, especially in areas with intense human activity, where human–carnivore coexistence tends to be difficult.

Presently, the puma is the most widespread top predator and one of the most conflictive carnivores in Argentina [10,11]. Pumas were historically present throughout Argentina. Thanks to its ecological plasticity, this species is relatively tolerant to a gradient of anthropogenically modified landscapes [12,13]. Nevertheless, in the last two centuries, since the colonization by Europeans, hunting of wild prey and extensive conversion of natural habitat into ranchland and farmland have increased encroachment with humans and predation on livestock [14]. In this scenario, pumas were listed as varmints for many years by a national law in Argentina (Ley Nacional No. 4863). In the second half of the last century, this feline was extirpated from most of Patagonia and from large portions of the Pampas [15]. More recently, the advance of the agriculture frontier and illegal hunting have caused a decline in puma population numbers and distribution in northeastern Argentina [16] and the southern part of Buenos Aires province [17,18], where currently its hunting is illegal. Livestock depredation by pumas often leads to retaliatory responses by agro-pastoralists, including opposition to wildlife sanctuaries, resistance to reintroduction of extirpated predators in protected areas as well as direct puma persecution [13,19–21] whose final consequence include local population extinction [21,22].

Although social, cultural and behavioural factors influence the decision by people to persecute large predators [4,23,24], in rural areas where livestock is an important source of income, livestock losses affect local people's attitudes towards, and acceptance of large felids (e.g. [23,25–28]). Economic losses caused by large feline predation of domestic species have been documented worldwide (e.g. [28–34]), including in the Americas (e.g. [2,27,35–37]). However, the estimates of the effects of puma depredation are often too rough to enable an understanding of its importance for local economies. Additionally, only a few studies explored the effects of conflicts associated with livestock predation both for local people and for the predators suffering retaliations [6,26,36,38].

### 1.1 Objectives

The present study aimed to characterize and quantify puma–livestock interactions in a human-dominated landscape of central Argentina. Our results are intended to provide information fundamental for the elaboration of effective conflict mitigation and conservation management strategies to guarantee both puma preservation and the viability of ranching activities globally and in this region, where puma hunting is illegal. Specifically, we estimated the rate of puma predation on livestock (‘depredation’ henceforward), quantified the losses caused to livestock ranchers, identified spatial–temporal depredation patterns, and explored how husbandry practices relate to depredation. Additionally, we estimated puma mortality rates and investigated the effect of depredation on puma hunting by ranchers. To independently validate some of these estimates, we carried out a comparison between interview-based, landscape-scale data and information obtained from direct surveys in a sub-area.

## 2. Material and methods

### 2.1. Study area

**Figure 1.**
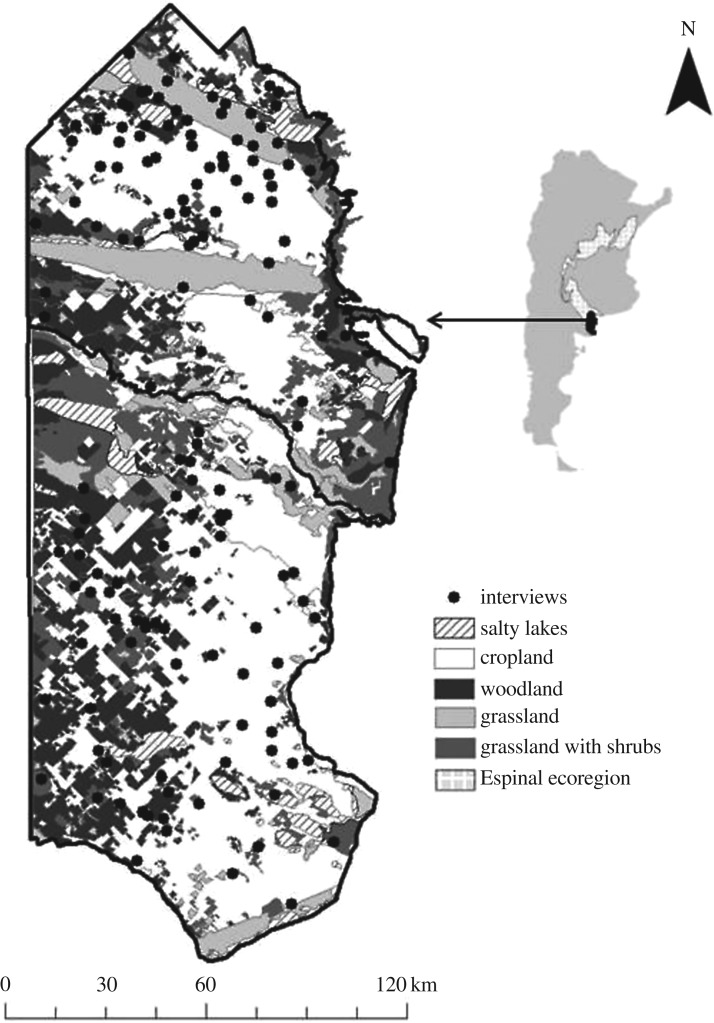
Maps showing the location of the study area (Villarino and Patagones counties) in Argentina and in the Espinal ecoregion, the habitat composition of the study area, and the locations of the interview sites. The black line shows the borders of the two counties, Villarino, in the north and Patagones, in the south.

We carried out our study in an area of 23 630 km^2^ located in the southern part of Buenos Aires Province, central Argentina, and corresponding to Villarino and Patagones counties (figure 1). The study area belongs to the Argentinean Espinal ecoregion. Located at an average of 700 m.a.s.l., this ecoregion is mostly flat. Climate is semiarid and the mean annual temperature is 15.3°C. The annual precipitation varies from 350 to 500 mm and falls mainly in spring and autumn [39]. The natural vegetation is characterized by xerophytic deciduous woodlands (dominated by *Prosopis caldenia*), prairies intermixed with natural patches of spontaneous scrub vegetation (also called grasslands with shrubs) and grassland prairies [40]. The Espinal is still inhabited by a diverse vertebrate community, including several carnivores and a unique array of herbivores [41]. However, this region has experienced a marked transformation during the last decades due to the intensification of crop cultivation and ranching activities, which are the primary sources of income [42]. The ongoing logging to create arable fields led to a habitat loss and fragmentation process that converted the original landscape into a mosaic of croplands with patches of natural vegetation [42]. Overgrazing has caused widespread degradation of soil and vegetation [43].

Although the two counties included in our study area have similar sizes, they show several differences that make their comparison meaningful. Biogeographically, Villarino is characterized by more closed xerophilous shrubby and spiny vegetation than Patagones where shrubby steppe is the dominant vegetation. Villarino also has a greater proportion of taller *Prosopis caldenia* woodlands.

According to the most recent official census (2015, Servicio Nacional de Sanidad y Calidad Agroalimentaria), the two counties also differed largely in the characteristics of livestock farming in the study period. Villarino had a greater number of smaller properties (i.e. ranches) than Patagones. On average, Villarino ranches had a greater quantity of cattle than Patagones, whereas ranches tended to focus more on sheep in Patagones (electronic supplementary material, appendix A).

### 2.2. Data collection and analyses

Data collection lasted from 2007 to 2015 and was based on interviews with local inhabitants and direct inspection of depredation sites. To obtain data on puma–livestock conflict and the perceptions and attitudes of local people towards the puma, we personally visited a stratified random sample of ranches and interviewed farmers and ranchers who worked there. Stratification was based on the habitat types available in the study area (cropland, grassland, grassland with shrubs, and woodland). Semi-structured interviews were conducted by two or three researchers in each case as face-to-face informal dialogues in order to create a friendly interaction between interviewers and interviewee and obtain honest answers. To maximize the honesty of the answers obtained by ranchers, we stated clearly at the beginning of each interview that we were from a non-governmental organization and that all information provided would be treated anonymously. At the end of the interview, a standard questionnaire, comprising both open and closed questions, was completed. To avoid spatial autocorrelation, we selected interview sites that were at least 4–5 km apart ([18]; figure 1). A range of data about the presence of key vertebrate species, their habitat association and respondents' perceptions towards them were recorded, but we focused here on questions regarding depredation by pumas and ranchers responses to it. Because interviews did not follow a rigid scheme and we wanted the ranchers to feel comfortable, some of them did not answer to all the questions and, as a consequence, sample sizes may vary across topics.

To estimate the financial losses caused by puma depredation, we used an average body weight of each type of livestock and its average annual market value in Argentine Pesos and multiplied this value by the number of livestock heads lost. Finally, to standardize results we applied the average Argentine Pesos/USD rate of exchange of the year when the loss was recorded. Additionally, we used a Chi-square test and the Jacobs index of selection [44] to compare the proportions of cattle and sheep killed by pumas to those available in each county and test the hypothesis that sheep were preferred over cattle [45,46].

Also, we tested the hypothesis resulting from previous studies on big feline–livestock conflicts [46–48] that the implementation of livestock husbandry practices, such as nocturnal corralling, would reduce predation rates. For this analysis, we limited our study to ranches that bred only sheep, because local ranchers believe that cattle are less vulnerable to puma predation and because they adopt extensive, free ranging breeding for cattle.

From 2013 to 2015, during 24 months we surveyed intensively an area of Patagones county (electronic supplementary material, appendix B) to validate the information obtained by interviews by personally checking all puma depredation events and puma kills reported by local ranchers. This area covered 513 km^2^ and was largely covered by woodlands (49.6%), followed by grassland with shrubs (23.7%), grassland (19.5%), and cropland (7.2%) and comprised 12 ranches; all of them bred cattle and four also raised sheep. Sheep totalled 970 heads (1.89 sheep km^−2^). The exact number of cattle heads was unknown but was much greater than that of sheep (greater than 2000 heads).

With the data recorded in this sub-area, we also tested the hypothesis that puma depredation was associated with sites and times of the day in which the risk of encountering humans was low [45,46,49]. Particularly, we predicted that depredation events on livestock would occur (i) in areas far from rancher houses, (ii) mostly at night-time, and (iii) in proximity of areas with dense vegetation cover (woodlands and grassland with shrubs). To test these predictions, we calculated the distances from each depredation site to the nearest house as well as to the closest patches of woodland and grassland with shrubs. The *t*-test was subsequently used to compare these distances to those calculated for 15 points randomly placed inside the corrals where sheep were present. The number of random points was equal to that of the predation events. To analyse habitat characteristics in relation to depredation sites, we applied a buffer of 100 m to the plots used by sheep as well as to the depredation sites and then used the proportional habitat composition of these two groups to calculate the value of the Jacobs index of selection for each habitat [44]. The values of this index vary from −1 (indicative of total avoidance) to +1 (indicative of complete preference). Habitat variables were calculated using ArcGIS (ESRI 2010. ArcGIS Desktop: Release 10. Redlands, CA: Environmental Systems Research Institute). Statistical analyses were performed using R v. 3.3.3 [50].

## 3. Results

### 3.1. Characterization of interviewee and ranches

We completed 111 and 102 interviews in Villarino and Patagones counties, respectively. The average age of respondents was 49.6 years (s.d. = 12.7 years; *n* = 178) and most (85.8%) were males. The sizes of the properties we surveyed varied greatly in both counties (table 1). In Patagones, most of the ranches kept herds of both sheep and cattle. Whereas ranches breeding only sheep were rare in Patagones, they were relatively common in Villarino (table 1).

**Table 1.** Size and proportions of ranches breeding sheep and cattle in the two counties of central Argentina (Villarino: *n* = 102; Patagones: *n* = 99) where puma–livestock interactions were surveyed.

	Villarino	Patagones
median property size (size range)	7 km^2^ (0.25–312 km^2^)	12 km^2^ (0.14–200 km^2^)
ranches breeding cattle and sheep	51.4% (*n* = 53)	69.7% (*n* = 69)
ranches breeding only cattle	22.3% (*n* = 22)	26.3% (*n* = 26)
ranches breeding only sheep	26.2% (*n* = 27)	4% (*n* = 4)

### 3.2. Livestock losses

**Figure 2.**
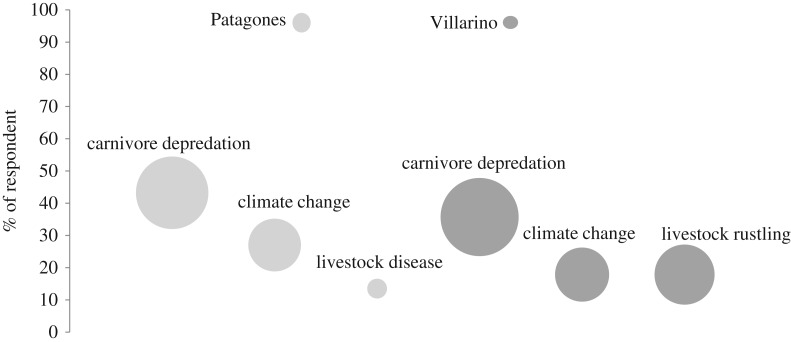
Main causes of livestock loss identified by local ranchers in the counties of Villarino and Patagones, Central Argentina. Bubble sizes are proportional to the percentage of respondents ranking a given factor as the most important cause, whereas their value on the vertical axis corresponds to the proportion of respondents listing that factor among the causes of livestock loss.

Carnivore predation, climate changes, rustling, and livestock diseases were the causes of livestock loss most frequently mentioned by ranchers (figure 2). Although variations were detected between the two counties (Villarino, *n* = 24 interviews; Patagones, *n* = 28), depredation was consistently considered the most important cause of livestock loss (figure 2).

Livestock losses were more frequent (*χ*^2^ = 7.3, d.f. = 2, *p* < 0.05) in Villarino than Patagones (46.6%, *n* = 48 and 35.4%, *n* = 35, respectively). In Villarino depredation was greater in mixed ranches than ranches breeding only sheep or cattle, whereas the highest frequency of puma attacks was reported by ranchers breeding only sheep in Patagones (table 2).

**Table 2.** Proportion of different types of ranches affected by puma depredation based on ranchers' reports for the year previous to the interview in the central Argentina counties of Villarino and Patagones.

	Villarino	Patagones
mixed ranches (cattle and sheep)	56.6% (*n* = 30)	44.9% (*n* = 31)
ranches breeding only sheep	44.4% (*n* = 12)	50% (*n* = 2)
ranches breeding only cattle	26% (*n* = 6)	7.7% (*n* = 2)

**Figure 3.**
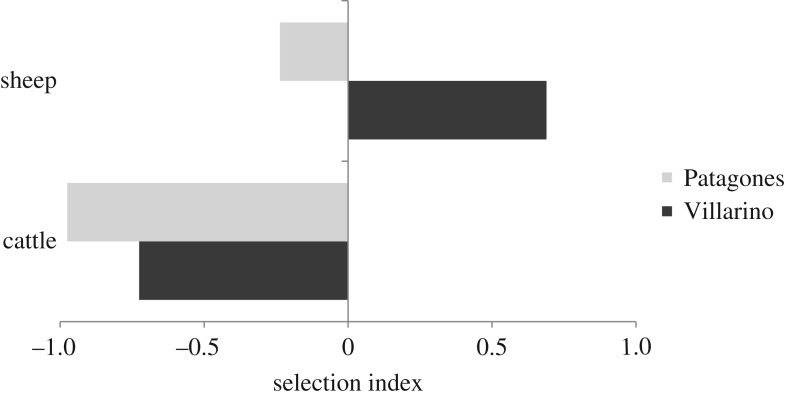
Puma selection (Jacobs index of selection) of livestock in Villarino (*n* = 46 ranches) and Patagones (*n* = 44). Positive values indicate that depredation was greater than expected based on livestock availability.

In both counties, the proportion of cattle and sheep killed by pumas differed from that expected based on their relative abundances (sheep: *χ*^2^ = 518.08, d.f. = 1, *p* < 0.05; cattle: *χ*^2^ = 24.9, d.f. = 1, *p* < 0.05). Predation was greater than expected only for sheep in Villarino, whereas in Patagones predation on sheep was essentially in accordance with their availability. Cattle depredation was less than expected in both counties (figure 3).

On average, the numbers and proportions of livestock killed as well as the economic losses attributed to pumas tended to be greater in Villarino than Patagones (table 3). However, depredation losses varied considerably among ranches. In Villarino, the ranges of losses were 0.1–51.9% for sheep and 2.1–3.3% for cattle in relation to living stock. In Patagones, sheep losses ranged from 0.7 to 86.7%, while predation on cattle was recorded in only one ranch and corresponded to 0.4% of its stock.

**Table 3.** Per ranch losses and economic damage caused by puma predation on livestock in two counties of central Argentina.

	Villarino		Patagones	
	cattle	sheep	cattle	sheep
number of ranches	14	27	14	30
median number of heads lost (mean ± s.d.)	0 (7.5 ± 22.6)	0 (45.1 ± 161.5)	0 (0.14 ± 0.53)	7 (15.6 ± 30.6)
% of heads lost to living stock	1.0	10.4	0.1	3.3
median economic loss (mean ± s.d., in USD)	0 (560 ± 1687.3)	0 (1560.4 ± 6466.4)	0 (5.3 ± 19.9)	315.8 (703.8 ± 1 382.8)

If we consider only those ranches where puma depredation occurred, the financial losses averaged 3398.4 USD per ranch per year (median = 541.4 USD, s.d. = 8729.4 USD, *n* = 17) for Villarino and 1059.5 USD (median = 541.4 USD, s.d. = 1587.1 USD, *n* = 20) in Patagones. Although the economic losses varied largely across individual ranches, the proportion of animals lost tended to be greater in ranches with less livestock (table 3 and electronic supplementary material, appendix C). In both counties the majority of ranches underwent losses smaller than 1000 USD (76.5%, *n* = 13, in Villarino; 75%, *n* = 15, in Patagones; electronic supplementary material, appendix C).

### 3.3. Ranchers' attitudes

In Villarino, 67.3% of all the respondents (*n* = 52) hunted pumas; 47.6% (*n* = 21) admitted that they had killed pumas in the previous 5 years and they estimated to have killed at least 57 individuals in total (11.4 pumas yr^−1^). Snares (34.6%; *n* = 9) were the most common hunting techniques used in this county, but people also frequently used fire arms, occasionally combined with dogs (26.9%; *n* = 7), and live-baited traps (26.9%; *n* = 7). In Patagones, 82.5% of the respondents (*n* = 57) reported that they used to hunt pumas and 29% (*n* = 31) had killed pumas in the previous 5 years. As a result, a minimum of 34 pumas were killed in that period (6.8 pumas yr^−1^). Fire arms (45.7%; *n* = 32) and snares (31.4%; *n* = 22) were the most commonly mentioned techniques to hunt pumas in Patagones.

Retaliation for depredation was not the only reason for hunting pumas. Only 31.9% (*n* = 47) of the ranchers who killed pumas had suffered livestock losses due to predation in Patagones compared to 77.8% (*n* = 36) for Villarino.

### 3.4. Mitigation measures adopted by ranchers

In Villarino, 57.1% of the ranches with sheep (*n* = 35) enclosed their sheep at night and 3.8% used guard animals (dogs and donkeys). Of the ranches sheltering sheep in enclosures during the night (*n* = 20), 80% suffered puma depredation. The ranches using corrals reported the loss of 8.7 ± 11.29 sheep ranch^−1^ (median = 5 sheep ranch^−1^) or 1.64% of the total stock (*n* = 5870 sheep), whereas those that did not adopt this practice lost 5.2 ± 13.2 sheep on average (median = 0 sheep ranch^−1^, 3.01% of the total, *n* = 1562 sheep). In Patagones, 53% of ranches with sheep (*n* = 33) used night enclosures to protect livestock and no one used guard animals. Of the ranches sheltering sheep in enclosures during the night, 42.8% (*n* = 14) suffered puma depredation. The ranches that used sheep enclosures endured the mean loss of 10.5 ± 13.1 livestock heads (median = 6 sheep), which corresponded to 1.22% of the total (*n* = 7880 sheep), whereas those without night enclosures lost an average of 22.9 ± 44.4 sheep (median = 8 sheep, 6.5% of the total, *n* = 4591 sheep).

In Villarino, the control of carnivore populations was mentioned most frequently (60%) as the most effective form to alleviate losses, followed by changes in livestock management (26.6%), and economic compensations (13.3%) (*n* interviewees = 14). The reduction of carnivore populations was considered the most effective measure to reduce depredation (66.6%) also in Patagones (*n* = 20), followed by change of livestock management (26.6%) and monetary compensation from the government (6.6%).

### 3.5. Direct inspection of kill sites

Of the ranches where kills were directly inspected (*n* = 12), 41.7% suffered depredation by puma in the intensively surveyed area, but this rate was 75% for the ranches which kept sheep and cattle (*n* = 4) and 16.7% for those with cattle only (*n* = 8). The same skew towards sheep was observed in the numbers of depredation events and of animals killed. Of predation events, 73.3% (*n* = 11) were on sheep and the remaining 26.7% (*n* = 4) were on calves (less than 12 months old). This was the only cattle age class predated by pumas in this area. If we consider all predated individuals (*n* = 42), 90.5% were sheep and the remaining 9.5% calves. Thus, puma killed 3.9% of all sheep present and 3.45 sheep and 1 calf per event, on average. Depredation rates were 4.09 individuals yr^−1^ per 100 km^2^ (21 individuals yr^−1^ and 0.08 individuals km^−2^). If only sheep are considered, depredation rates were 3.7 sheep yr^−1^ per 100 km^2^.

The total economic loss caused by puma depredation during the entire period of 24 months was 2466 USD. The loss was equivalent to 1916 USD for the 3 ranches with sheep (or 319.3 USD per ranch per year) and to 550 USD for the 2 ranches that endured predation on cattle (137.5 USD per ranch per year). However, if we consider the 12 ranches included in this area, the mean yearly loss was equivalent to 102.8 USD.

**Figure 4.**
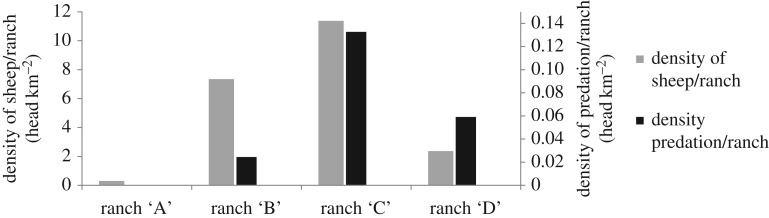
Total sheep density and density of sheep predated by pumas in the four ranches breeding sheep in an area of Patagones county of central Argentina where all depredation events were recorded and directly inspected. ‘B’ ranch was the only one that applied night enclosure as mitigation measure against puma depredation.

The intensity of depredation did not appear to be related to sheep density in this sub-area (figure 4). Only one of the ranches (ranch B) used to enclose sheep in pens for the night. During the survey period, this ranch kept a herd of 300 sheep and recorded a single depredation event (figure 4), which occurred during a night when sheep were not corralled.

**Figure 5.**
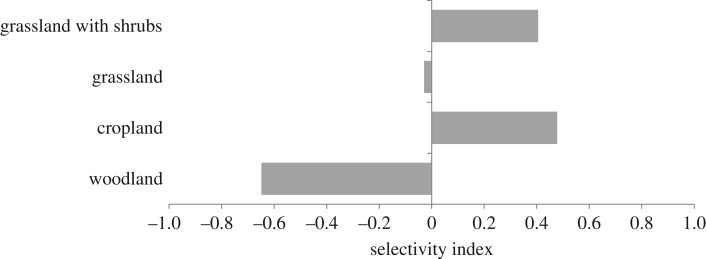
Puma habitat selection (Jacob index of selection) in the sheep predation sites in an intensively monitored area of the Patagones county of central Argentina.

Based on the data obtained from our direct inspections of kill sites (*n* = 11), depredation events occurred most in cropland (36.5%), followed by grassland (25.9%), grassland with shrubs (22.8%) and woodland (14.9%). In contrast, woodland was the most abundant habitat (45%) in the corrals where sheep were kept (total area = 70.6 km^2^), followed by grassland (27%), cropland (16.9%), and grassland with shrubs (11.1%). This suggests that pumas selected cropland and grassland with shrubs and avoided woodlands when preying upon sheep in this area (figure 5).

The distances from depredation sites to the nearest rancher's house ranged from 0.73 to 6.69 km (average = 2.89 km) and did not differ from those expected based on randomly selected points (*t*-test: *t* = 10.99, *p* > 0.05). Within this range, 86.7% of depredation events occurred at more than 1 km from the ranchers' houses. The average distance from depredation sites was 0.64 km (range: 0–1.8 km) to the nearest woodland patch and 0.14 km (range: 0–0.37 km) to the closest patch of grassland with shrubs and did not differ from random points (*t* = 0.86, *p* > 0.05 for woodlands; *t* = 0.84, *p* > 0.05, for grassland with shrubs).

As predicted, all depredation events occurred during the night-time.

### 3.6. Puma mortality rates

Based on the answers from interviewed ranchers, we calculated a mortality rate for each county. In Villarino 1.56 pumas yr^−1^ per 100 km^2^ were killed while this rate was 1.1 pumas yr^−1^ per 100 km^2^ in Patagones. Puma mortality rates averaged 1.36 pumas yr^−1^ per 100 km^2^ on the basis of the directly confirmed mortality events (*n* = 14) in the intensively surveyed area.

## 4. Discussion

Livestock depredation can inflict high losses to local livelihoods, especially in poor communities [4,51,52]. However, to our knowledge, this is the first large-scale study that evaluated socio-economic and ecological aspects of puma–livestock conflicts, simultaneously quantifying large feline predation on livestock and human killing of pumas in a temperate region of South America across a wide range of property sizes, livestock herd sizes, and management practices. Thus, our results contribute to fill an important gap of knowledge about puma–livestock interactions in Latin America [14,53]. We found that the conflict between pumas and livestock producers in central Argentina is very intense (see also [54]) and that ranchers respond by killing pumas at alarming rates. Irrefutably, pumas caused a relatively large amount of losses and, regardless of depredation, were perceived by ranchers as a major threat to their livelihoods. These results are in accordance with the scarce information available on puma–livestock conflicts in southern South America. Half of the 165 livestock producers interviewed in the Argentinean province of Santa Cruz reported puma predation on their stock [55]. On four large sheep ranches in southern Patagonia, ranchers reported annual sheep losses to pumas as 3–9% of sheep stocks and killed up to 15 pumas every year on their properties [14]. Similar levels of losses were reported in Chilean Patagonia, where puma killing was also common [56].

Although puma depredation was widespread in our study area, affecting 35.4–46.6% of ranches in the two counties, the proportions of animals killed were minimal in cattle (0.06–1%) and 3.3–10.4% in sheep. Additionally, even when only the ranches affected by depredation were included and if we assume that the losses were not exaggerated by ranchers (which may not be the case, see [57,58]), the financial losses endured by the ranchers averaged 2134 USD per ranch and year and were less than 1000 USD in 75–76.5% of the cases. This amount is likely to represent a very large proportion of a family income in some developing countries. However, as *per capita* Gross Domestic Product (GDP) in Argentina was 10 332 USD in 2010 [59] and a great majority of the families affected by predation were certainly above the average level of income for this country, we can conclude that the damage inflicted by puma predation to regional livestock industry was economically limited.

On the other hand, it is important to consider that some ranchers, typically small sheep farmers from Villarino county, carried a higher burden of the overall costs of depredation and experienced levels of depredation so high that they were likely to be economically critical. This is confirmed by the fact that some interviewees, in particular individual producers that experienced multiple depredations or excessive killing, abandoned sheep farming because it was no longer profitable. A similar pattern of sheep breeding abandonment from unacceptably high scales of puma predation was previously observed elsewhere [37]. Because research on risk perception indicates that people focus on maximal events rather than the average [60], this uneven distribution of depredation costs among ranchers may unduly influence their intolerance for predators, with negative effects on carnivore conservation as it was reported for wolves (*Canis lupus*; [61–63]).

Although indirect losses (e.g. abortion, weight loss, milk production decrease) potentially linked with predation [2,35] were not frequently mentioned by interviewees, we did not measure them. We advise that future studies on this subject should attempt to quantify indirect losses, to avoid underestimating the effect of puma predation on livestock.

Although puma preference for sheep and goats [45,46] is probably a major reason of unequal distribution of livestock losses among ranchers, we also found a strong county-related variation in this preference. In addition to the fact that puma predation was much greater than expected in Villarino, we also found that both the frequency of puma attacks and the impact of depredation tended to be greater in Villarino than Patagones. Consistently, recent puma hunting was more pervasive and more connected to depredation levels in Villarino. A similar discrepancy in attitudes towards snow leopards (*Panthera uncia*) between two contiguous sites in the Indian Himalaya was attributed to differences in the economic value of livestock in these two areas [64]. We argue that in our study ranchers in Villarino were less tolerant basically because they experienced greater levels of depredation.

Several studies have identified landscape variables, such as proportions of forest in proximity of ranches and distances to forests and water sources, as predictors of conflicts with carnivores [27,45,47,49]. Although most of the predation sites we inspected were in proximity of forest patches and far from houses, we failed to find any effect of distances from dense vegetation and houses on the intensity of depredation by puma. However, the frequency of puma attacks was greater than expected in patches of croplands and grasslands with shrubs. Because grassland with shrubs was the habitat preferred by pumas in the same study area [18,65], this result is in agreement with the hypothesis that the distribution of puma habitat would largely explain sheep predation risk [53,66,67]. Although these findings need confirmation, they also suggest that failure to consider the values expected on the basis of livestock use of habitat may guide to misleading conclusions on the effect of habitat on predation risk.

### 4.1. Conflict mitigation

Although the effect of nocturnal enclosures on puma predation appears contradictory [2,37], our data from interviews and kill site inspections indicate that corralling sheep at night may reduce the extent of depredation by pumas. This is not surprising given that, as expected for a region where pumas are heavily hunted [5,45], pumas have nocturnal habits and their depredation concentrated at night-time in our study area and that freely grazing livestock is particularly vulnerable to hunting [2,7]. The reduction in losses we found in ranches that used nocturnal enclosures supports the conclusions of other studies in Latin America [45,46,47,68] that livestock management, specifically nocturnal corralling, should prove effective to alleviate depredation by puma, especially if corrals are correctly designed and built. Nevertheless, we found that only few ranchers mentioned the improvement of livestock husbandry as a potentially effective tool to reduce losses, while a majority of ranchers claimed that lethal control is the most adequate measure. Ranchers may not modify their practices to prevent predation on livestock and prefer to continue hunting predators because they do not have necessary knowledge, resources or readiness, or because of their negative attitude towards carnivores and conservation [13,45]. Approval of killing predators is expected to increase when predators live on private properties, as in our study, and when social norms or beliefs reinforce predator killing as a societal good [24,69,70]. In our study area, ranchers frequently mentioned depopulation of rural areas as a major cause of increasing conflicts with carnivores [54]. If this is the case, a more widespread and efficient implementation of husbandry practices requiring greater labour may be difficult because it would imply substantial economic investments [71].

Whereas puma killing is illegal in our study area as in the rest of Buenos Aires province, since 1995 a bounty system is in place to mitigate puma depredation in the neighbouring province of Rio Negro and in two additional Patagonian provinces. These bounties are considerable by Argentinean economic standards, especially for low-paid ranch workers, and up to 2000 puma bounties have been paid per year in these three provinces [14]. In spite of this extremely large number of pumas killed, complaints about puma predation on livestock continue and puma attacks are still considered a major cause of livestock loss in Patagonia [72]. Although the effect of puma control on the livestock industry has never been studied, the extent to which carnivore removal reduces predation losses remains equivocal [73]. Several studies have shown that increasing efforts to control predator populations did not produce reductions in livestock depredation [19,73]. Although our mortality estimates are crude and should be treated with caution, they indicate that far more pumas were killed by ranchers in our study area (1.1–1.56 individuals per 100 km^2^) than those legally removed by the bounty system in the Patagonian provinces (*ca* 0.3 individuals per 100 km^2^; [14]). It is difficult to imagine that a puma population in the rangelands of central Argentina could sustain such high mortality rates over the long term. Unsuitability of most habitats to pumas in this territory [18] and rarity of pumas in comparison with other carnivores [54,65] support this conclusion and suggest careful monitoring of this top predator and its ecological functions in central Argentina.

## 5. Conclusion

As most big felines, the puma is a globally iconic but locally problematic species. In the context of conflict, the long-term conservation of large carnivores can only be achieved if the strategies that are developed and implemented outweigh the local costs incurred [4]. Thus, the information we provided is relevant to understanding the dynamics of human–puma conflicts globally and supports the development of meaningful and applicable management plans for their mitigation locally.

In our study, puma depredation concentrated on sheep and a significant level of losses was endured by a relatively small number of sheep ranchers, which probably shaped intolerant attitudes towards pumas. Because puma depredation typically occurred at night, nocturnal corralling of sheep, especially if enclosures are properly built, guarded by dogs and/or located next to inhabited houses, appears to be a simple and effective measure to alleviate depredation. Although more data are needed, it is also possible that livestock losses could be reduced if ranchers avoid keeping sheep in the habitats locally preferred by pumas. Further improvement may be obtained if other measures, such as guard dogs, financial compensation or insurance schemes are also adopted, as it is unlikely that a single method will function for ranches differing largely in their characteristics. However, the process of rural areas abandonment represents a potential limitation of the implementation of these measures and should be accounted for in the decision-making process.

Because reduced availability of natural prey may increase large feline depredation [74,75] and wild prey may serve to buffer livestock from puma predation [76,77], land management that facilitates a greater abundance of wild prey may also potentially decrease depredation by pumas in the rangelands of central Argentina.

Notwithstanding, we argue that conflict mitigation in central Argentina is not only a question of reducing actual damage but also of increasing tolerance [23,78–81]. Thus, in agreement with [82–84], we conclude that the implementation of awareness programmes targeting local people and aiming to increase their knowledge about carnivores and their role in ecosystems is important to favour long-term coexistence of pumas and ranching activities in central Argentina. Finally, we surmise that strong conflicts between pumas and local people are exacerbated by the lack of response from the government, which neither compensates losses to ranchers nor offers a forum for their complaints. The success of human–puma coexistence strategies will require a range of actions that are both effective and guarantee the full engagement of local stakeholders in wildlife management decisions acceptable [51].

## Supplementary Material

Farming activities (Appendix A)

## Supplementary Material

Map of habitat composition (Appendix B)

## Supplementary Material

Stock and ecnomic losses (Appendix C)

## Supplementary Material

Semi-structured inteview sheet; Predation record data sheet; Kill site database

## Data accessibility

Our data are deposited at Dryad (http://dx.doi.org/10.5061/dryad.k5904) [85].

## References

[1] Woodroffe R 2000 Predators and people: using human densities to interpret declines of large carnivores. Anim. Cons. 3, 165–173. (doi:10.1111/j.1469-1795.2000.tb00241.x)

[2] Mazzolli M, Graipel ME, Dunstone N 2002 Mountain lion depredation in southern Brazil. Biol. Cons. 105, 43–51. (doi:10.1016/S0006-3207(01)00178-1)

[3] Baker PJ, Boitani L, Harris S, Saunders O, White PCL 2008 Terrestrial carnivores and human food production: impact and management. Mammal Rev. 38, 123–166. (doi:10.1111/j.1365-2907.2008.00122.x)

[4] Dickman A, Marchini S, Manfredo M 2013 The human dimension in addressing conflict with large carnivores. In Key topics in conservation biology, vol. 2 (ed. Macdonald DW), pp. 110–126. Oxford, UK: Wiley-Blackwell (528 pp).

[5] Ohrens O, Treves A, Bonacic C 2015 Relationship between rural depopulation and puma-human conflict in the high Andes of Chile. Environ. Cons. 43, 24–33. (doi:10.1017/S0376892915000259)

[6] Treves A, Karanth KU 2003 Human-carnivore conflict and perspectives on carnivore management worldwide. Cons. Biol. 17, 1491–1499. (doi:10.1111/j.1523-1739.2003.00059.x)

[7] Zarco-González MM, Monroy-Vilchis O, Rodríguez-Soto C, Urios V 2012 Spatial factors and management associated with livestock predations by *Puma concolor* in Central Mexico. Hum. Ecol. 40, 631–638. (doi:10.1007/s10745-012-9505-4)

[8] Estes JA et al. 2011 Trophic downgrading of planet Earth. Science 333, 301–306. (doi:10.1126/science.1205106) 21764740 10.1126/science.1205106

[9] Ripple WJ, Estes JA, Beschta RL, Wilmers CC, Ritchie EG, Hebblewhite M 2014 Status and ecological effects of the world's largest carnivores. Science 343, 1241484 (doi:10.1126/science.1241484) 24408439 10.1126/science.1241484

[10] Donadio E, Novaro AJ, Buskirk SW, Wurstten A, Vitali MS, Monteverde MJ 2010 Evaluating a potentially strong trophic interaction: pumas and wild camelids in protected areas of Argentina. J. Zool. 280, 33–40. (doi:10.1111/j.1469-7998.2009.00638.x)

[11] Llanos R, Llanos MB, Travaini A 2016 ¿Qué ves cuando me ves? El puma (*Puma concolor*) y su representación en los medios de prensa escrita de Patagonia Argentina. Interciencia 41, 16–22.

[12] Foster RJ, Harmsen BJ, Doncaster CP 2010 Habitat use by sympatric jaguars and pumas across a gradient of human disturbance in Belize. Biotropica 42, 724–731. (doi:10.1111/j.1744-7429.2010.00641.x)

[13] Quiroga VA, Noss AJ, Paviolo A, Boaglio GI, Di Bitetti MS 2016 Puma density, habitat use and conflict with humans in the Argentine Chaco. J. Nat. Cons. 31, 9–15. (doi:10.1016/j.jnc.2016.02.004)

[14] Walker S, Novaro A 2010 The World's Southernmost Pumas in Patagonia and the Southern Andes. In Cougar: ecology and conservation. (eds Hornocker MG, Negri S), pp. 91–103. Chicago, USA: University of Chicago (304 pp).

[15] Parera A. 2000 Los mamíferos de la Argentina y la región austral de Sudamérica. Buenos Aires, Argentina: El Ateneo (452 pp).

[16] De Angelo C, Paviolo A, Di Bitetti M 2011 Differential impact of landscape transformation on pumas (*Puma concolor*) and jaguars (*Panthera onca*) in the Upper Paraná Atlantic Forest. Divers. Distr. 17, 422–436. (doi:10.1111/j.1472-4642.2011.00746.x)

[17] De Lucca ER 2010 Presencia del puma (*Puma concolor*) y conflicto con el hombre en las pampas argentinas. Nótulas Faunísticas 48, 1–17.

[18] Caruso N, Guerisoli M, Luengos Vidal EM, Castillo D, Casanave EB, Lucherini M 2015 Modelling the ecological niche of an endangered population of *Puma concolor*: first application of the GNESFA method to an elusive carnivore. Ecol. Model. 297, 11–19. (doi:10.1016/j.ecolmodel.2014.11.004)

[19] Graham K, Beckerman AP, Thirgood S 2005 Human-predator-prey conflicts: ecological correlates, prey losses and pattern of management. Biol. Cons. 122, 159–171. (doi:10.1016/j.biocon.2004.06.006)

[20] Amit R, Gordillo-Chávez EJ, Bone R 2013 Jaguar and puma attacks on livestock in Costa Rica. Hum. Wildl. Interact. 7, 77–84.

[21] Pia MV 2013 Evaluación del conflicto entre los carnívoros tope y productores ganaderos colindantes al Parque Nacional Quebrada del Condorito, Sierras Grandes de Córdoba, Argentina. Nótulas Faunísticas 2, 1–10.

[22] Hoogesteijn R, Hoogesteijn AL 2014 Antipredation strategies for cattle ranches in Latin America: a guide. Campo Grande, Brazil: Panthera (63 pp).

[23] Zimmermann A, Walpole MJ, Leader-Williams N 2005 Cattle ranchers' attitudes to conflicts with jaguars in the Pantanal of Brazil. Oryx 39, 406–412. (doi:10.1017/S0030605305000992)

[24] Treves A, Bruskotter JT 2014 Tolerance for predatory wildlife. Science 34, 476–477. (doi:10.1126/science.1252690) 10.1126/science.1252690 24786065

[25] Quigley HB, Crawshaw PG Jr 1992 A conservation plan for the jaguar *(Panthera onca*) in the Pantanal region of Brazil. Biol. Cons. 61, 149–157. (doi:10.1016/0006-3207(92)91111-5)

[26] Mishra C 1997 Livestock depredation by large carnivores in the Indian trans-Himalaya: conflict perceptions and conservation prospects. Environ. Cons. 24, 338–343. (doi:10.1017/S0376892997000441)

[27] Michalski F, Boulhosa RLP, Faria A, Peres CA 2006 Human–wildlife conflicts in a fragmented Amazonian forest landscape: determinants of large felid depredation on livestock. Anim. Cons. 9, 179–188. (doi:10.1111/j.1469-1795.2006.00025.x)

[28] Babrgir S, Farhadinia MS, Moqanaki EM 2017 Socio-economic consequences of cattle predation by the endangered Persian leopard *Panthera pardus saxicolor* in a Caucasian conflict hotspot, northern Iran. Oryx 51, 124–130. (doi:10.1017/S0030605315000903)

[29] Stahl P, Vandel J, Ruette S, Coat L, Coat Y, Balestra L 2002 Factors affecting lynx predation on sheep in the French Jura. J. Appl. Ecol. 39, 204–216. (doi:10.1046/j.1365-2664.2002.00709.x)

[30] Patterson B, Kasiki S, Selempo E, Kays R 2004 Livestock predation by lions (*Panthera leo*) and other carnivores on ranches neighboring Tsavo National Parks, Kenya. Biol. Cons. 119, 507–516. (doi:10.1016/j.biocon.2004.01.013)

[31] Wang S, Macdonald DW 2006 Livestock predation by carnivores in Jigme Singye Wangchuck National Park, Bhutan. Biol. Cons. 129, 558–565. (doi:10.1016/j.biocon.2005.11.024)

[32] Van Bommel L, Bij de Vaate M, De Boer W, De Iongh H 2007 Factors affecting livestock predation by lions in Cameroon. Afr. J. Ecol. 45, 490–498. (doi:10.1111/j.1365-2028.2007.00759.x)

[33] Marker LL, Boast LK 2015 Human-wildlife conflict 10 years later: lessons learned and their application to cheetah conservation. Hum. Dimens. Wildl. 20, 302–309. (doi:10.1080/10871209.2015.1004144)

[34] Minnie L, Boshoff AF, Kerley GI 2015 Vegetation type influences livestock predation by leopards: implications for conservation in agro-ecosystems. Afr. J. Wildl. Res. 45, 204–214. (doi:10.3957/056.045.0204)

[35] de Azevedo F, Murray D 2007 Evaluation of potential factors predisposing livestock to predation by jaguars. J. Wildl. Manage. 113, 2379–2386. (doi:10.2193/2006-520)

[36] Amador-Alcalá S, Naranjo EJ, Jiménez-Ferrer G 2013 Wildlife predation on livestock and poultry: implications for predator conservation in the rainforest of south-east Mexico. Oryx 47, 243–250. (doi:10.1017/S0030605311001359)

[37] Schulz F, Printes RC, Oliveira LR 2014 Depredation of domestic herds by pumas based on farmer's information in Southern Brazil. J. Ethnobiol. Ethnomed. 10, 73 (doi:10.1186/1746-4269-10-73) 25318598 10.1186/1746-4269-10-73 PMC4271476

[38] Dar NI, Minhas R A, Zaman Q, Linkie M 2009 Predicting the patterns, perceptions and causes of human–carnivore conflict in and around Machiara National Park, Pakistan. Biol. Cons. 142, 2076–2082. (doi:10.1016/j.biocon.2009.04.003)

[39] Cabrera AL, Willink A. 1980 Biogeografía de América Latina. Washington, DC: O.E.A. Serie Monográfica No 4. (122 pp).

[40] Cano E, Movia C 1967 Utilidad de la fotointerpretación en la cartografía de comunidades vegetales del bosque de caldén (*Prosopis caldenia*). INTA. Instituto Botánico Agrícola. La vegetación de la república Argentina 8, 1–44.

[41] Barquez RM, Díaz MM, Ojeda RA. 2006 Mamíferos de Argentina: Sistemática y Distribución. Tucuman, Argentina: Sociedad Argentina para el estudio de los Mamíferos (SAREM) (354 pp).

[42] Canevari P, Blanco DE, Bucher E, Castro G, Davison I. 1998 Los Humedales de la Argentina. Clasificación, situación actual, conservación y legislación, vol. 46 Buenos Aires, Argentina: Wetlands International (208 pp).

[43] Distel RA 2016 Grazing ecology and the conservation of the Caldenal rangelands, Argentina. J. Arid Environ. 134, 49–55. (doi:10.1016/j.jaridenv.2016.06.019)

[44] Jacobs J 1974 Quantitative measurement of food selection: a modification of the forage ratio and Ivlev's electivity index. Oecologia 14, 413–417. (doi:10.1007/BF00384581) 28308662 10.1007/BF00384581

[45] Soto-Shoender JR, Giuliano WM 2011 Predation on livestock by large carnivores in the tropical lowlands of Guatemala. Oryx 269, 7–19.

[46] Zarco-González MM, Monroy-Vilchis O, Alaníz J 2013 Spatial model of livestock predation by jaguar and puma in Mexico: conservation planning. Biol. Cons. 159, 80–87. (doi:10.1016/j.biocon.2012.11.007)

[47] Polisar J, Maxit I, Scognamillo D, Farrell L, Sunquist ME, Eisenberg JF 2003 Jaguars, pumas, their prey base, and cattle ranching: ecological interpretations of a management problem. Biol. Cons. 109, 297–310. (doi:10.1016/S0006-3207(02)00157-X)

[48] Boulhosa RLP, Azevedo FCC 2014 Perceptions of ranchers towards livestock predation by large felids in the Brazilian Pantanal. Wildl. Res. 41, 356–365. (doi:10.1071/WR14040)

[49] Palmeira F, Crawshaw P, Haddad C, Ferraz K, Verdade L 2008 Cattle depredation by puma (*Puma concolor*) and jaguar (*Panthera onca*) in central western Brazil. Biol. Cons. 141, 118–125. (doi:10.1016/j.biocon.2007.09.015)

[50] R Core Team. 2013 R: a language and environment for statistical computing. Vienna, Austria: R Foundation for Statistical Computing.

[51] Sillero-Zubiri C, Laurenson MK 2001 Interactions between carnivores and local communities: conflict or co-existence? In Carnivore conservation (eds Gittleman JL, Funk SM, Macdonald DW, Wayne RK), pp. 282–312. Cambridge, UK: Cambridge University Press.

[52] Thirgood S, Woodroffe R, Rabinowitz A 2005 The impact of human–wildlife conflict on human lives and livelihoods. In People and wildlife: conflict or coexistence? (eds Woodroffe R, Thirgood S, Rabinowitz A), pp. 13–26. Cambridge, UK: Cambridge University Press (506 pp).

[53] Daniel Kissling W, Fernández N, Paruelo JM 2009 Spatial risk assessment of livestock exposure to pumas in Patagonia, Argentina. Ecography 32, 807–817. (doi:10.1111/j.1600-0587.2009.05781.x)

[54] Luengos Vidal E, Guerisoli M, Caruso N, Casanave E, Lucherini M 2017 Conflictos con el puma en el sur del Espinal argentino. In Conflictos humanos-felinos América Latina, pp. 363–375. Colombia: Instituto de Investigación de Recursos Biológicos Alexander von Humboldt (489 pp).

[55] Travaini A, Zapata SC, Martínez-Peck R, Delibes M 2000 Percepción y actitud humanas hacia la predación de ganado ovino por el zorro colorado (*Pseudalopex culpaeus*) en Santa Cruz, Patagonia Argentina. Mastozool. Neotrop. 7, 117–129.

[56] Franklin WL, Johnson WE, Sarno RJ, Iriarte JA 1999 Ecology of the Patagonia puma *Felis concolor patagonica* in southern Chile. Biol. Cons. 90, 33–40. (doi:10.1016/S0006-3207(99)00008-7)

[57] Gipson PS, Ballard WB, Nowak RM 1998 Famous North American wolves and the credibility of early wildlife literature. Wildl. Soc. Bull. 26, 808–816.

[58] Fritts SH 1982 *Wolf depredation on livestock in Minnesota* (No. 145). US Fish and Wildlife Service.

[59] World Bank Official Data. 2016 *Gross Domestic Product* (GDP) in Argentina. See http://data.worldbank.org/indicator/NY.GDP.PCAP.CD?locations=AR (accessed December 2016).

[60] Lehmkuhler J, Palmquist G, Ruid D, Willging B, Wydeven A 2007 Effects of wolves and other predators on farms in Wisconsin: beyond verified losses. Pub-ER-658. Madison, WI: Wisconsin Wolf Science Committee, Wisconsin Department of Natural Resources. See http://dnr.wi.gov/files/pdf/pubs/er/er0658.pdf.

[61] Naughton-Treves L, Grossberg R, Treves A 2003 Paying for tolerance: the impact of livestock depredation and compensation payments on rural citizens' attitudes toward wolves. Cons. Biol. 17, 1500–1511. (doi:10.1111/j.1523-1739.2003.00060.x)

[62] Gazzola A, Avanzinelli E, Bertelli I, Tolosano A, Bertotto P, Musso R, Apollonio M 2007 The role of the wolf in shaping a multi-species ungulate community in the Italian western Alps. Ital. J. Zool. 74, 297–307. (doi:10.1080/11250000701447074)

[63] Muhly TB, Musiani M 2009 Livestock depredation by wolves and the ranching economy in the Northwestern US. Ecol. Econ. 68, 2439–2450 (doi:10.1016/j.ecolecon.2009.04.008)

[64] Bagchi S, Mishra C 2006 Living with large carnivores: predation on livestock by the snow leopard (*Uncia uncia*). J. Zool. 268, 217–224. (doi:10.1111/j.1469-7998.2005.00030.x)

[65] Caruso N, Lucherini M, Fortin D, Casanave EB, Moreira N 2016 Species-specific responses of carnivores to human-induced landscape changes in central Argentina. PLoS ONE 11, e0150488 (doi:10.1371/journal.pone.0150488) 26950300 10.1371/journal.pone.0150488 PMC4780817

[66] Torres SG, Mansfield TM, Foley JE, Lupo T, Brinkhaus A 1996 Mountain lion and human activity in California. Wild. Soc. Bull. 24, 451–460.

[67] Tortato FR, Layme VMG, Crawshaw PG, Izzo TJ 2015 The impact of herd composition and foraging area on livestock predation by big cats in the Pantanal of Brazil. Anim. Cons. 18, 539–547. (doi:10.1111/acv.12207)

[68] Crawshaw PG 2004 Depredation of domestic animals by large cats in Brazil. Hum. Dimens. Wildl. 9, 329–330. (doi:10.1080/10871200490505774)

[69] Treves A, Naughton-Treves L 1999 Risk and opportunity for humans coexisting with large carnivores. J. Hum. Evol. 36, 275–282. (doi:10.1006/jhev.1998.0268) 10074384 10.1006/jhev.1998.0268

[70] Marchini S, Macdonald DW 2012 Predicting ranchers’ intention to kill jaguars: case studies in Amazonia and Pantanal. Biol. Cons. 147, 213–221. (doi:10.1016/j.biocon.2012.01.002)

[71] Rey Benayas JM, Martins A, Nicolau JM, Schulz J 2007 Abandonment of agricultural land: an overview of drivers and consequences. CAB Rev. Perspect. Agriculture Vet. Sci. Nat. Resources 2, 1–14. (doi:10.1079/PAVSNNR20072057)

[72] Llanos R, Travaini A, Montanelli S, Crespo E 2014 Estructura de edades de pumas (*Puma concolor*) cazados bajo el sistema de remoción por recompensas en Patagonia. *¿Selectividad u oportunismo en la captura?* Ecol. Austral. 24, 311–319.

[73] Berger KM 2006 Carnivore-livestock conflicts: effects of subsidized predator control and economic correlates on the sheep industry. Cons. Biol. 20, 751–761. (doi:10.1111/j.1523-1739.2006.00336.x) 10.1111/j.1523-1739.2006.00336.x 16909568

[74] Loveridge AJ, Wang SW, Frank LG, Seidensticker J 2010 People and wild felids: conservation of cats and management of conflicts. In Biology and conservation of wild felids (eds Macdonald DW, Loveridge AJ), pp. 161–195. Oxford, UK: Oxford Univ. Press (784 pp).

[75] Khorozyan I, Ghoddousi A, Soofi M, Waltert M 2015 Big cats kill more livestock when wild prey reaches a minimum threshold. Biol. Cons. 192, 268–275. (doi:10.1016/j.biocon.2015.09.031)

[76] Acosta-Jamett G, Gutiérrez JR, Kelt DA, Meserve PL, Previtali MA 2016 El Niño-Southern Oscillation drives conflict between wild carnivores and livestock farmers in a semiarid area in Chile. J. Arid Environ. 26, 76–80. (doi:10.1016/j.jaridenv.2015.08.021)

[77] Hiller TL, Mcfadden-Hiller JE, Jenkins SR, Belant JL, Tyre AJ 2015 Demography, prey abundance, and management affect number of cougar mortalities associated with livestock conflicts. J. Wildl. Manage. 79, 978–988. (doi:10.1002/jwmg.913)

[78] Marker LL, Mills MGL, Macdonald DW 2003 Factors influencing perceptions and tolerance toward cheetahs (*Acinonyx jubatus*) on Namibian farmlands. Cons. Biol. 17, 1–9. (doi:10.1046/j.1523-1739.2003.02077.x)

[79] Jackson RM, Wangchuk R 2004 A community-based approach to mitigating livestock depredation by snow leopards. Hum. Dimens. Wildl. 9, 1–16. (doi:10.1080/10871200490505756)

[80] Gusset M, Maddock AH, Gunther GJ, Szykman M, Slotow R, Walters M, Somers MJ 2008 Conflicting human interests over the re-introduction of endangered wild dogs in South Africa. Biodivers. Cons. 17, 83–101. (doi:10.1007/s10531-007-9232-0)

[81] Garcia-Alaniz N, Naranjo EJ, Mallory FF 2010 human-felid interactions in three mestizo communities of the Selva Lacandona, Chiapas, Mexico: benefits, conflicts and traditional uses of species. Human Ecol. 38, 451–457. (doi:10.1007/s10745-010-9322-6)

[82] Conforti VA, Cesar Cascelli de Azevedo F 2003 Local perceptions of jaguars (*Panthera onca*) and pumas (*Puma concolor)* in the Iguaçu National Park area, south Brazil. Biol Cons. 111, 215–221. (doi:10.1016/S0006-3207(02)00277-X)

[83] Carvalho EA, Pezzuti JC 2010 Hunting of jaguars and pumas in the Tapajós–Arapiuns Extractive Reserve, Brazilian Amazonia. Oryx 44, 610–612. (doi:10.1017/S003060531000075X)

[84] Engel MT, Vaske JJ, Bath AJ, Marchini S 2016 Predicting acceptability of jaguars and pumas in the Atlantic Forest, Brazil. Hum. Dimens. Wildl. 21, 427–444. (doi:10.1080/10871209.2016.1183731)

[85] Guerisoli MM, Luengos Vidal E, Franchini M, Caruso N, Casanave EB, Lucherini M 2017 Data from: Characterization of puma--livestock conflicts in rangelands of central Argentina. Dryad Digital Repository (http://dx.doi.org/10.5061/dryad.k5904) 10.1098/rsos.170852 PMC5749996 29308228

