# Supplementary material for: Characterization of puma–livestock conflicts in rangelands of central Argentina
Source: R Soc Open Sci. 2017 Dec 6;4(12):170852. doi: 10.1098/rsos.170852 (PMC5749996; doi:10.1098/rsos.170852)
Supplement: Farming activities (Appendix A) [file rsos170852supp1.docx]

Appendix A. Main characteristics of the farming activities in the two counties of Villarino and Patagones representing our study area in central Argentina.

|  | Villarino | Patagones |  |
| --- | --- | --- | --- |
| County size (km^2^) | 10 051 | 13 579 |  |
| Number of ranches | 882 | 627 |  |
| Mean ranch size (km^2^) | 11.4 | 21.7 |  |
| Number of sheep heads | 46 683 | 248 261 |  |
| Sheep heads/km^2^ | 4.6 | 18.3 |  |
| Sheep heads/ranch | 52.9 | 396 |  |
| Number of cattle heads | 355 663 | 255 711 |  |
| Cattle heads/km^2^ | 35.4 | 18.8 |  |
| Cattle heads/ranch | 403.2 | 407.8 |  |
