# Supplementary material for: Characterization of puma–livestock conflicts in rangelands of central Argentina
Source: R Soc Open Sci. 2017 Dec 6;4(12):170852. doi: 10.1098/rsos.170852 (PMC5749996; doi:10.1098/rsos.170852)

Appendix B. Map showing the habitat composition in an area of Patagones county of central Argentina where all depredation events were recorded and directly inspected.


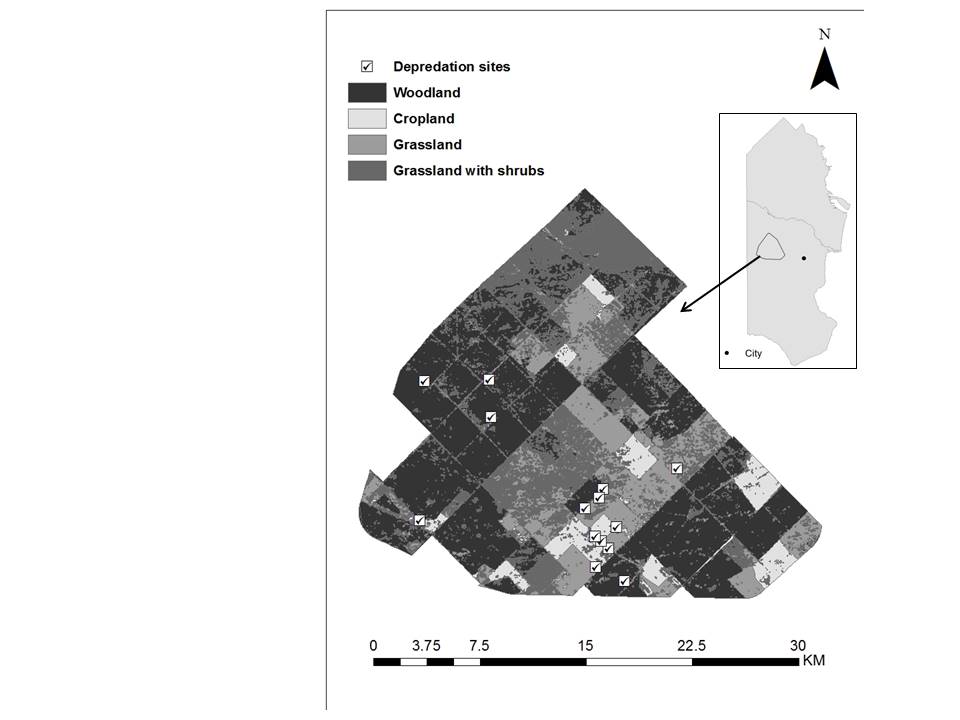

Supplement: Map of habitat composition (Appendix B) [file rsos170852supp2.docx]
