# Supplementary material for: Characterization of puma–livestock conflicts in rangelands of central Argentina
Source: R Soc Open Sci. 2017 Dec 6;4(12):170852. doi: 10.1098/rsos.170852 (PMC5749996; doi:10.1098/rsos.170852)
Supplement: Stock and ecnomic losses (Appendix C) [file rsos170852supp3.docx]

Appendix C. Stock and economic losses (in USD) caused by puma depredation on livestock for individual ranches in Villarino county (a) and Patagones county (b).

a)

b)
