# Supplementary material for: Characterization of puma–livestock conflicts in rangelands of central Argentina
Source: R Soc Open Sci. 2017 Dec 6;4(12):170852. doi: 10.1098/rsos.170852 (PMC5749996; doi:10.1098/rsos.170852)
Supplement: Semi-structured inteview sheet; Predation record data sheet; Kill site database [file rsos170852supp4.doc]

NUMBER OF DATA SHEET……..

**INTERVIEW TO LOCAL PEOPLE ON CARNIVORES IN THE ESPINAL**

**DATE: _____________________ INTERVIEWER: ___________________________________________**

**RANCH NAME: __________________________________ COORDINATES: ___________________________________________**

Grassland

Bushland

Water body

**LOCATION: ______________________________________HABITAT (general):**

cropland

dunes

other

pastures

**INTERVIEWEE DATA:**

NAME: ___________________________ SEX: _________AGE: ______ NUMBER OF MEMBERS IN THE FAMILY: __________

**LEVEL OF EDUCATION:**

Primary school: Secondary school: High School: University:

**ACTIVITY**: Ranch owner: Ranch employee: Housewife:

Municipal employee: Other (specify):________________________

Livestock heads owned: cattle ________ sheep ________ pigs _______ chickens ________ other________

**SIZE OF PROPERTY:**  ___________________ (ha)

Lives in the area Lives in the ranch How long he/she has been living in the region?

**MANAGEMENT**

Type of Livestock: extensive intensive “feed lot”

Changes management seasonally? YES NO

If so, which changes he/she made?___________________________________________________

____________________________________________________________________________________________

Birth: species _____________ in enclosure YES NO Temporally concentrated YES NO

Enclose livestock at night? YES NO Uses guard animals? YES NO If so, what species? ___________

Provides livestock with mineral or food supplements? YES NO

Order the following causes of mortality / loss of livestock (1= most important; 6= least important):

| Deseases |  |
| --- | --- |
| Livestock robbery |  |
| Drought |  |
| Depredation |  |
| Lack of food |  |
| Other_____________________ |  |

**PERCEPTIONS:**

Does consider that the puma is harmful in the region?

Much - - Little - - Nothing - -

Pumas get close to the main house in the ranch? YES NO

How many livestock has lost because of puma depredation?

|  | **During the last year** | | |
| --- | --- | --- | --- |
|  | **N. of heads** | **Argentinean pesos** |  |
| Cattle |  |  |  |
| Calf |  |  |  |
| Sheep |  |  |  |
| Lamb |  |  |  |
| Chicken |  |  |  |
| Other |  |  |  |

The attacks are usually more common during? (afternoon, night, dawn or she/he does not know)

____________________________

**ATTITUDES:**

What does the interviewee do when a puma causes damage?

# Tries to kill it Tries to scare it away Changes livestock management Nothing

If she/he tries to kill it, what method is used?

Active hunting Snares/leg-hold trap Box-trap Poison Hunting Dogs Other: _____________________________

Notes (changes in livestock management, etc.)

| Has he/she ever hunted pumas?  A lot: A little: Never:    Has he/she hunted pumas in the last 5 years? YES NO  If so, how many?___________ |
| --- |

**COMMENTS OR CONCERNS THAT THE INTERVIEWEE WANTS TO SHARE**


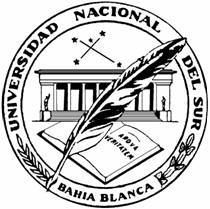

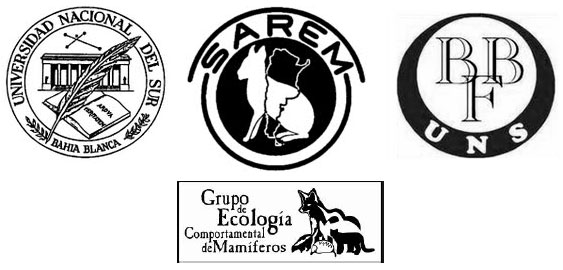
 **PUMA PROJECT: DEPREDATION**

Date: __________ ID predation: __________ Predation date: ________ Ranch name:_______________

X: ______________ Y: ______________ Operators: _________________________________

Prey species: __________________ Number of preys: _________ Age: _____________

Depredation moment (afternoon, night, etc.): ___________ Habitat: _____________

Prey covered: YES____ NO _____ Vegetation Type: _________________________

Puma hunted: _______ Method used: __________________ Dead puma ID: _______________

Was killed prey in a corral?: ________ Guard animal: YES___NO____ What species? _________

Birth period? _________________

Canine distance in the prey (1): _______________ Canine distance in the prey (2): ____________

Signs and depredation dynamic:

Describe / draw the area of attack and if there was dragging (distance, habitat, etc.):

Describe how the puma fed on the prey (what parts were eaten):

Notes:

Data base Kill site

| **ID_Pred** | **X** | **Y** | **Ranch name** | **Prey** | **quantity** | **Day time** |
| --- | --- | --- | --- | --- | --- | --- |
| Pred_16 | 496312 | 5581274 | Cordoba | sheep | 1 | Night |
| Pred_22 | 494263 | 5582246 | Haure | sheep | 1 | Night |
| Pred_15 | 495188 | 5583579 | Haure | sheep | 1 | Night |
| Pred_14 | 494638 | 5584069 | Haure | sheep | 3 | Night |
| Pred_21 | 494252 | 5584410 | Haure | lamb | 6 | Night |
| Pred_11 | 495741 | 5585058 | Haure | sheep | 2 | Night |
| Pred_10 | 481841 | 5585518 | Rauch F. | sheep | 1 | Night |
| Pred_25 | 494814 | 5587751 | Haure | sheep | 1 | Night |
| Pred_12 | 500044 | 5589215 | Ilgner | calf | 1 | Night |
| Pred_23 | 482184 | 5595360 | Mochas | calf | 1 | Night |
| Pred_29 | 486761 | 5595464 | Mochas | calf | 1 | Night |
| Pred_30 | 486904 | 5592814 | Las mochas | calf | 1 | Night |
| Pred_31 | 494510 | 5587126 | Lauquen | sheep | 7 | Night |
| Pred_32 | 494510 | 5587126 | Lauquen | lamb | 10 | Night |
| Pred_33 | 493518 | 5586367 | Haure | sheep | 5 | Night |
